# Presence of C1-Inhibitor Polymers in a Subset of Patients Suffering from Hereditary Angioedema

**DOI:** 10.1371/journal.pone.0112051

**Published:** 2014-11-04

**Authors:** Daniel Elenius Madsen, Søren Hansen, Jørgen Gram, Anette Bygum, Christian Drouet, Johannes Jakobsen Sidelmann

**Affiliations:** 1 University of Southern Denmark, Institute of Public Health, Unit for Thrombosis Research, Esbjerg, Denmark; 2 University of Southern Denmark, Institute of Molecular Medicine, Cancer and Inflammation Research, Odense C, Denmark; 3 Hereditary Angioedema Centre Denmark, Department of Dermatology and Allergy Centre, Odense University Hospital, Odense C, Denmark; 4 University Joseph Fourier Grenoble, National Reference Centre for Angioedema, Grenoble, France; University of Thessaly, Faculty of Medicine, Greece

## Abstract

Hereditary angioedema (HAE) is a potentially life-threatening disease caused by mutations in the gene encoding the serine protease inhibitor (serpin) C1 inhibitor (C1-inh). The mutations cause decreased functional plasma levels of C1-inh, which triggers unpredictable recurrent edema attacks. Subjects suffering from HAE have been classified in type I patients with decreased functional and antigenic levels of C1-inh, and type II patients with decreased functional but normal antigenic C1-inh levels. However, a few reports have demonstrated that some mutations cause C1-inh polymerization in vitro, and it is speculated that C1-inh polymers may exist in patient plasma, challenging the current classification of HAE patients. To investigate the presence of C1-inh polymers in patient plasma samples, we developed an immunological method, where monoclonal antibodies produced against polymerized C1-inh were applied in native PAGE western blotting. Using this approach we analyzed genuine plasma samples from 31 Danish HAE families, and found that plasma samples from three genotypically distinct HAE type I families (classified upon C1-inh plasma concentrations) contained C1-inh polymers. Identical C1-inh polymerization phenotypes were observed in four affected family members from one of these families. Genotyping of the families revealed that the polymerogenic mutations of two families were located in proximity to the reactive center loop insertion site in C1-inh (p.Ile271Thr and p.Ser258_Pro260del),and one mutation affected helix C (p.Thr167Asn). In conclusion, we demonstrate that C1-inh polymers are present in the plasma of a subgroup of HAE type I patients.

## Introduction

Hereditary angioedema (HAE) is a life-threatening autosomal dominant inherited disease, caused by mutations in the SERPING1 gene encoding the serine protease inhibitor (serpin) C1-inhibitor (C1-inh) [1]–[4]. Plasma samples from patients with HAE are characterized by decreased functional levels of C1-inh [5]. Traditionally, patients have been classified in two subtypes: HAE type I patients are characterized by low functional and antigen plasma levels of C1-inh, whereas HAE type II patients are characterized by low functional, but normal or increased antigen C1-inh plasma levels [6], [7]. This classification has however been challenged by observations of intermediary HAE types, that can arise, when small amounts of dysfunctional C1-inh is present in the blood stream [8]. As no evidence regarding clinical consistencies between the type I and type II patients have been observed, this classification describes as such, only the biochemical profile of HAE patients (and not the presentation of HAE itself). Both types of patients suffer from episodic swellings, where bradykinin (BK) is suspected to play a central role [4], [9]. The edema formation is primarily caused by a transient increased BK release from high molecular weight kininogen (HMWK). The BK release is mediated by uncontrolled activation of the coagulation factor XII (FXII) dependent kallikrein kinin system [10].

C1-inh circulates in plasma in a stressed high energetic metastable conformation, which is characterized by a reactive center loop (RCL) protruding from the central part of the serpin. The amino acid sequence of the RCL serves as a bait region for a limited number of proteases. When a protease recognizes and cleaves the P1–P1′ scissile bond in the RCL, the RCL domain inserts into the central beta-sheet A of C1-inh together with the covalently attached protease. After cleavage C1-inh obtains a low energetic stable conformation, and the protease is irreversibly inhibited [11]. Polymerized C1-inh represents another stable and low energetic conformation, which can be attained upon mutations in the *SERPING1* gene. A few studies have *in vitro* addressed the ability of mutated C1-inh to form polymers [8], [12]–[14]. The studies focused on distinct mutations resulting in C1-inh polymerization, and recombinantly expressed mutated C1-inh proteins were utilized to demonstrate polymerization of the C1-inh *in vitro*. For example Zahedi et al. demonstrated that the C1-inh mutant C1-inh-Ta (p.Lys251_del) had an increased propensity to polymerize when expressed recombinantly [13]. One group did observe a multimeric form of C1-inh in fractions from sucrose gradient centrifugation of a patient plasma sample, and this suggested that C1-inh polymers might exist in the plasma of HAE patients [12].

Extracellular serpin polymers have been observed in other diseases involving mutations in serpin encoding genes. A classic example hereof is the presence of α_1_-antitrypsin polymers in lung lavage of patients suffering from the Z-mutation (p.Glu342Lys) in the α_1_-antitrypsin encoding gene [15].

The clinical relevance of C1-inh polymers in the plasma of HAE patients remains hitherto uncertain, and therefore we aimed to elucidate the presence and nature of C1-inh polymers in plasma from HAE patients.

## Materials and Methods

### Plasma and genotyping of HAE patients

EDTA and CPDA plasma samples were collected from 75 Danish HAE patients representing 31 families and 34 healthy individuals (BD Vacutainer K_2_EDTA tubes, Becton Dickinson, Plymouth, UK and Vacuette CPDA tubes, Greiner Bio-One, Wemmel, Belgium, respectively). The tubes were centrifuged at 2000 g for 20 minutes. The plasma was aliquoted and stored at −80°C. An EDTA plasma pool was made by pooling plasma samples from 30 healthy individuals. Screening for C1-inh polymers was performed by analyzing one plasma sample representative of each family (n = 31). Plasma samples from all individuals of the families tested positive for C1-inh polymers by the screening procedure were subsequently analyzed. Genotypes were identified as previously reported. [1].

### Preparation of native C1-inh stock

Two ampules of Berinert (CSL Behring, King of Prussia, PA, USA) each containing 500 international units of C1-inh were dissolved in 20 mL PBS. The dissolved content was transferred to Spectra/Por Dialysis Membrane MWCO 6–8,000 (Spectrumlabs, Rancho Dominquez, CA, USA) and dialysed thrice against 5 L of PBS pH 7.4 for a total of 72 hours at 4°C. Protein concentration was measured by UV absorption at 280 nm (molar extinction coefficient: 0.382 mL mg^−1^ cm^−1^) to 6.4 mg/mL, and aliquots were stored at −80°C.

### Preparation of C1-inh polymers

C1-inh aliquots (6.4 mg/mL) were thawed at 37°C for 15 minutes. Fifty µL were transferred to PCR-tubes and heated at 65°C for 35 minuntes on a Peltier Thermal Cycler PTC-200 (MJ Research Inc., Waltham, MA, US). C1-inh polymers were frozen in 50 µL aliquots at −80°C immediately after preparation. These polymers are denoted pC1-inh.

### Preparation of low molecular weight C1-inh polymers

C1-inh polymers were produced as described above, with the modification of heating at 55°C for 35 min. These polymers were subsequently separated using gelfiltration chromatography on a 110 mL Superose 6 preparation grade column (GE Healthcare, Piscataway, NJ, US), and fractions containing low molecular weight polymers (LMW pC1-inh) were pooled. The LMW pC1-inh was concentrated using anion exchange chromatography on a 1 mL Resource Q column (GE Healthcare). The concentration of C1-inh was calculated using the molar extinctions coefficient for native C1-inh.

### Preparation of SDS-stable C1-inh polymers

A preparation pC1-inh (30.5 µM, 3.2 mg/mL) was incubated with 40 µM of bis-sulfosuccinimidyl-suberate (BS^3^) (Thermo Scientific Inc., Waltham, MA, US) in PBS, pH 7.4 for 35 minutes at 25°C with agitation. The reaction was quenched by incubation for 20 minutes with 50 mM Tris, and aliquots were immediately frozen at −80°C.

### Native- and SDS PAGE analysis of proteins

BS^3^ conjugated pC1-inh, pC1-inh and monomeric C1-inh were separated using native PAGE on 4–15% Mini-PROTEAN TGX precast gels (Bio-Rad, Hercules, CA, US) using Tris/Glycine buffer (Bio-Rad). Initial dilutions of the proteins were made in PBS, and prior to analysis samples were diluted 50% (v/v) in native PAGE samples buffer (Bio-Rad). Final protein content per lane of BS^3^ pC1-inh, and pC1-inh were 10 µg and for monomeric C1-inh it was 2 µg. The gel was processed for 105 minutes at 150 volts, and was visualized using silver staining according to Nesterenko et al [16].

SDS-stable BS^3^ conjugated pC1-inh, pC1-inh and monomeric C1-inh were separated using SDS-PAGE on 4–15% Mini-PROTEAN TGX precast gels using Tris/Glycine/SDS buffer (Bio-Rad). All initial dilutions were made in PBS, and subsequently a 50% (v/v) dilution was made in 2X Laemmli Buffer (Bio-Rad) before boiling at 96°C for 8 minutes. Samples of 0.13 µg protein were applied per lane and were processed for 90 minutes at 150 volts. The gel was visualized using silver staining and a molecular weight marker (HMW-SDS Marker Kit, GE Healthcare Life Sciences, Picastaway, NJ, US) was used to correlate mobility with MWs.

### Antibody production

MAbs against C1-inh polymers were produced essentially as described by Köhler and Milstein [17] and modified by Galfrè and Milstein [18]. Briefly, female BALB/c X NMRI mice were immunized subcutaneously with pC1-inh adsorbed onto Al(OH)_3_ and emulsified in Freund's Incomplete Adjuvant (Brenntag Biosector, Frederikssund, Denmark). Culture supernatant from the hybridomas was screened using MicroWell MaxiSorp plates (Thermo Fisher Scientific, Roskilde, Denmark) coated with 1 µg C1-inh polymers/mL. Wells with positive signals were cloned for monoclonality using the limited dilution method, and subsequently the cells were grown in serum free RPMI medium for large scale production. The antibodies were purified using MabSelect SuRe (GE Healthcare, Piscataway, NJ, USA) Protein A column as described by Ey et al. [19].

The animals euthanized for harvesting the spleens, were euthanized by trained personnel, by approved methods (cervical dislocation). The animals were bred and maintained at the central animal facility of the University of Southern Denmark, and there was daily supervision by trained animal technicians and a veterinarian. Any signs of poor health or compromised welfare were treated immediately, or the affected animals were euthanized. Prior to euthanasia animals were housed at a 12 hours dark/light cycle, with light from 6 am to 6 pm at 21°C+/−1°C, and a relative humidity between 45 and 65%. Animals were caged in macro room type III cages, with aspen wood shaped bedding material and Enviro-dri nesting material (Shepherd Speciality Papers, Richland, MI, US). Animals were fed ad libitum Altromin vial 1324 (Altromin Spezialfutter GmbH & Co. KG, Lage, Germany), and ad libitum tap water from drinking bottles. Animals had access to a tunnel as shelter.

Twenty-four monoclonal antibodies were produced and characterized with regard to their relative affinity towards C1-inh polymers in native PAGE WB. One antibody (12-27-15) was selected for further experiments based on its high affinity in this setting.

### Native PAGE WB analysis of proteins

pC1-inh, LMW pC1-inh, and C1-inh were loaded in amounts of 1 µg, 60 ng and 24 ng per well, respectively, on 4–15% Mini-PROTEAN TGX precast gels and separated by native PAGE as described above. Gels were blotted onto polyvinylidene fluoride membranes using the Trans-Blot Turbo Blotting System (Bio-Rad) with Trans-Blot Transfer Pack consumables (Bio-Rad) for 30 minutes at 25 volt. Membranes were blocked in PBS with 0.05% tween-20 (PBS-TW) containing 2.5% skimmed milk powder at 24°C for 45 minutes with agitation, and incubated overnight at 4°C with 2 µg MAb 12-27-15/mL PBS-TW. Membranes were washed thrice in PBS-TW, and incubated for 1 hour at 24°C in PBS-TW with horseradish peroxidase conjugated rabbit-anti-mouse-Ig diluted 1∶4000 (Zyma, Life Technologies, Invitrogen, Carlsbad, CA, US) in PBS-TW. Reactivity was visualized with 3-amino-9-ethylcarbazol and H_2_O_2_.

### Native PAGE WB analysis of patient samples and controls

Patient EDTA plasma samples were thawed at 24°C for 25 minutes, and diluted to 25% (v/v) in PBS buffer, and further diluted 50% (v/v) in native sample buffer (Bio-Rad), giving a final plasma dilution of 10% (v/v). pC1-inh (1 µg/lane), LMW pC1-inh (60 ng/lane), C1-inh (24 ng/lane) and the EDTA plasma pool (10% (v/v)) were used for controls. Patient samples and controls (pC1-inh, LMW pC1-inh and C1-inh) were analyzed as described above. CPDA plasma samples from all patients and EDTA plasma samples from 34 healthy individuals were also analyzed as described above. The effect of five repeated freeze/thaw cycles on the native PAGE WB phenotype of an EDTA plasma pool was investigated to assure that polymerization of C1-inh was not caused by freezing and thawing.

### Structural predictions

To visualize the location of the C1-inh mutations at protein level, a protein structure modelling was performed using Chimera 1.8.1 for Windows (UCSF, Resource for Biocomputing, Visualization and Informatics, San Francisco, CA, US). Each mutation was positioned on the model according to the coordinates given by the protein databank entry 1M6Q and 2OAY, which represent a homology model of the native C1-inh serpin domain and an X-ray crystallographic structure of latent C1-inh, respectively [20], [21].

### Ethics

All subjects gave informed written consent to blood sampling and mutational analysis, and the study was conducted in accordance with the Helsinki II Declaration and approved by the Regional Scientific Ethics Committee for Southern Denmark (Project-id: S-20110047) and the Danish Data Protection Agency (2008-58-0035).

Animal experiments were perform according to institutional guidelines, under the license issued by the Danish Animal Experiments Inspectorate April 12th, 2010 license number “2010/561-175”.

## Results

### Genotype of HAE patients

The *SERPING1* genotypes of the patients with each peptide mutant position are listed in Table 1 [1]. Each mutation was given a mutation number (Mut. no.), and the genotypes of five patients were unknown. These five patients were treated as five different genotypes. They were abbreviated G^1^–G^5^. Each individual patient was identified with an identification number (ID. no.).

**Table 1:** Mutational characteristics of patients.

Mut. no.	cDNA	Protein	ID. no.
1	c.1417G>A	p.Val451Met	34, 35, 61, 64, 65
2	c.551_685del	Del EX4	8, 9, 21, 22, 45, 60, 72, 82, 90, 98
3	c.1480C>T	p.Arg472X	12, 32, 62, 63
4	c.119_141dup	p.Thr26GlufsX39	19, 36, 59, 84, 91
5^II^	c.1397G>A	p.Arg444His	11, 16, 24, 89, 94
6	c.878T>C	p.Ile271Thr	5, 6, 28, 33
7	c.1-22-2A>G	Splice def.	14, 17, 39, 55, 66, 67, 71, 76, 77, 95, 100
8	c.143_144delCA	p.Thr26SerfsX9	30
9	c.437delT	p.Leu124TrpfsX2	3, 50, 51
10	c.1029+4delA	Splice def.	26, 37, 38, 47, 87, 101
11	c.668delA	p.Gln201ArgfsX10	43, 83
12	c.550G>A	p.Gly162Arg	29, 93
13	c.838:846del	p.Ser258_Pro260del	20
14	c.762_763delCA	p.Asn232LysfsX2	54
15	c.23dupT	X19 in signal pept.	80
16	c.795G>A	p.Trp243X	73
17	c.1381G>C	p.Ala439Pro	99
18	c.1279delC	p.Leu405CysfsX4	2
19	c.1427C>T	p.Pro454Leu	1
20	c.1030_1503del	Del EX7 and EX8	46
21	c.566C>A	p.Thr167Asn	23
22	c.1250-1G>A	Splice def.	31
G^1^	N/A	N/A	13
G^2^	N/A	N/A	92
G^3^	N/A	N/A	96
G^4^	N/A	N/A	97
G^5^	N/A	N/A	103

Grouping of patients according to *SERPING1* genotype. A mutation number (Mut. no.) was ascribed to each genotype. CDNA- and protein variants were numbered as by Bygum et al. [1]. G^1^–G^5^ represent four different patients with unknown genotypes. Mut. no. 5^II^ represents that patients bearing this mutation are classified HAE type II patients. All other patients are classified as HAE type I.

### Native- and SDS PAGE analysis of proteins

**Figure 1:**
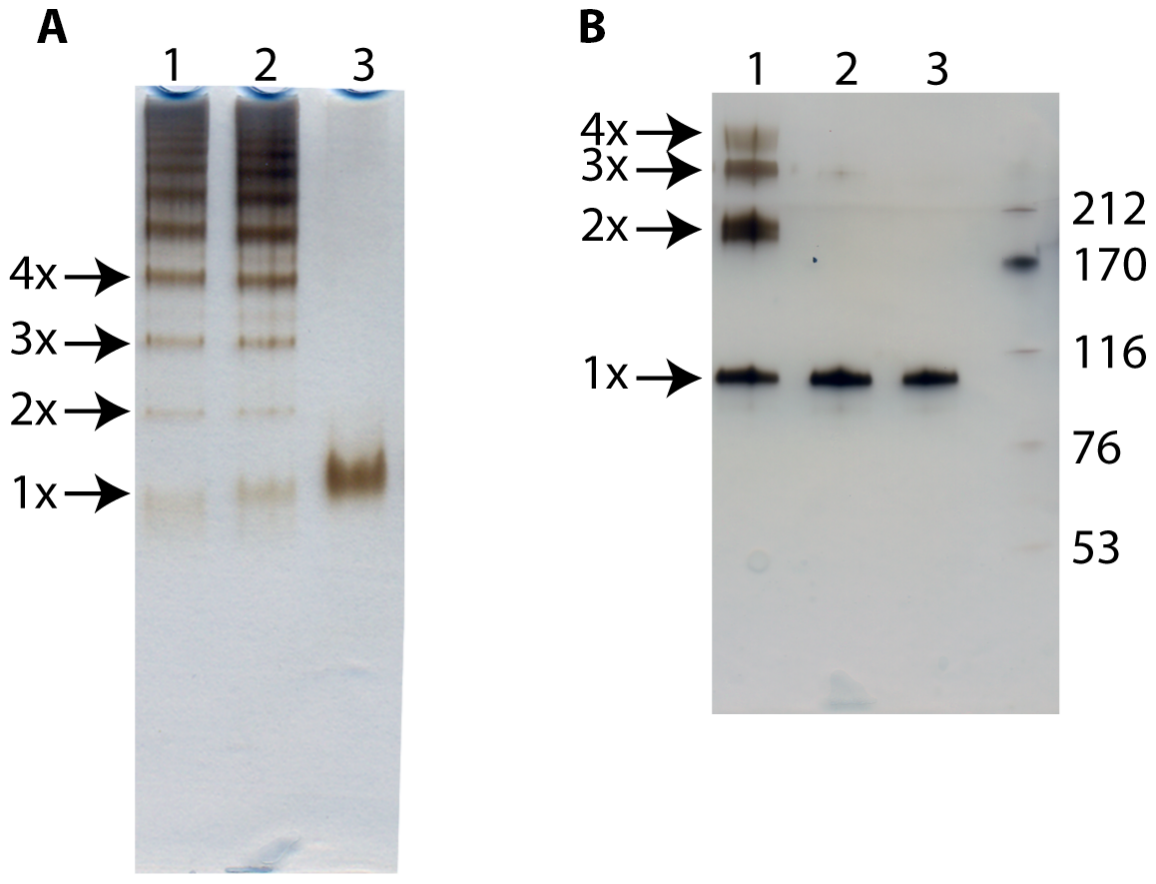
PAGE analysis of native and polymerized C1-inh. A) Native PAGE analysis of BS^3^ conjugated pC1-inh (lane 1), pC1-inh (lane 2) and native C1-inh (lane 3). Proteins were visualized using silver staining. B): SDS PAGE analysis of BS^3^ conjugated pC1-inh (lane 1), pC1-inh (lane 2), native C1-inh (lane3) and a molecular weight marker (lane M). Molecular weights of the marker proteins are depicted in kDa to the right. Proteins were visualized using silver staining.

Polymerization of C1-inh was visualized using native PAGE analysis (Fig. 1A). The pC1-inh preparation contained polymers of various sizes, and the electrophoretic mobility of pC1-inh remained unaffected by the BS^3^ conjugation. Trace amounts of monomeric C1-inh were observed in the two polymerized samples. The molecular weight of BS^3^ conjugated C1-inh polymers (Fig. 1B). corresponded to those anticipated for C1-inh polymers. pC1-inh was not SDS-stable, but dissolved to the monomeric form when boiled in the presence of SDS.

### Western blotting experiments

**Figure 2:**
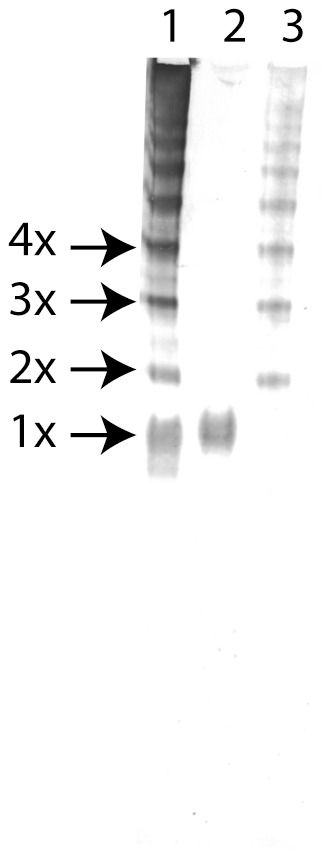
Native PAGE WB analysis of pC1-inh (lane 1), native C1-inh (lane 2) and LMW pC1-inh (lane 3). Visualized using MAb 12-27-15, HRP conjugated rabbit anti mouse IgG and 3-amino-9-ethylcarbazol and H_2_O_2_.

The reactivity of MAb 12-27-15 was tested in native PAGE western blotting against pC1-inh, monomeric C1-inh and LMW pC1-inh. Fig. 2 shows that MAb 12-27-15,recognizes all tested forms of C1-inh including: pC1-inh, native C1-inh and LMW pC1-inh.

### Native PAGE WB analysis of patient samples

**Figure 3:**
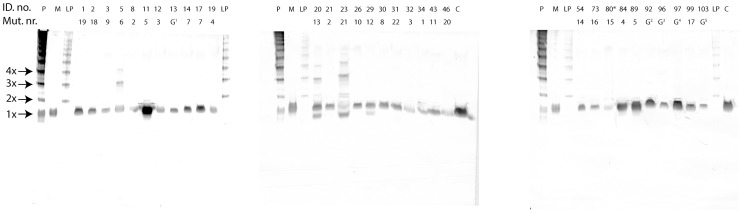
Native PAGE WB analysis of EDTA plasma samples from 31 HAE patients. “ID. no.” depicts in house patient number, “Mut. no.” depicts individual mutation numbers, “P” depicts C1-inh polymers formed at 65°C for 35 min, “M” depicts monomeric C1-inh, “LP” denotes low molecular weight C1-inh polymers formed using gel filtration and ion exchange chromatography, “C” depicts an EDTA plasma pool, “G” depicts that the genotype of the patient is unknown., “*” indicates that CPDA plasma was analyzed instead of EDTA plasma.

**Figure 4:**
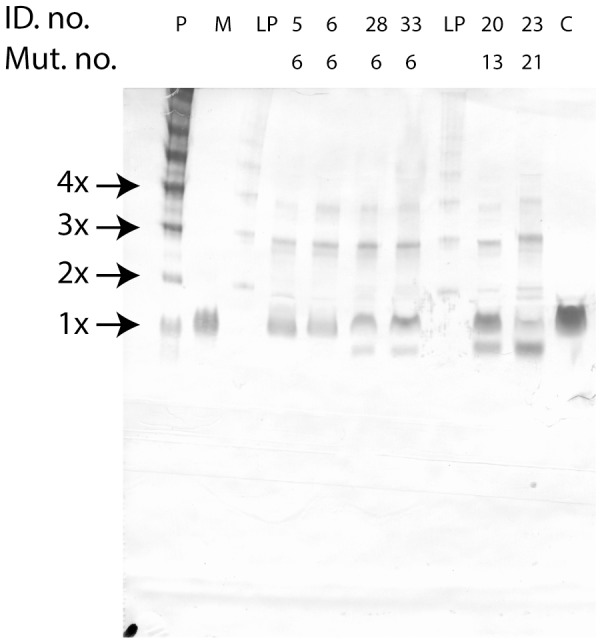
Native PAGE analysis of EDTA plasma samples from six HAE patients, representing three different genotypes. Nomenclature used as for figure 3.

Thirty-one patient EDTA plasma samples representing each HAE family were analyzed for the presence of C1-inh polymers using native PAGE WB (Fig. 3). Polymerized C1-inh was identified in plasma samples from patients with ID. no. 5, 6, 28 and 33 (Mut. no. 6), ID. no. 20 (Mut. no. 13) and ID. no. 23 (Mut. no. 21). The polymers ranged in size from dimers to tetramers, with only Mut. no. 21 giving rise to a tetrameric band. The dominant oligomeric species among the polymer positive patients was the trimeric band. The four patients carrying Mut. no. 6 were analyzed in parallel (Fig. 4), and displayed identical polymerization patterns, different from those observed for Mut. no. 13 and 21. In ID. nos. 28, 33, 20 and 23 a low molecular weight form of C1-inh was also observed. This band was not observed in ID. nos. 5 and 6, although these patients carry the same mutation as ID. no. 28 and 33. ID. no. 23 presented with a band between the mono- and dimeric species. This band might represent C1-inh in association with a protease.

**Figure 5:**
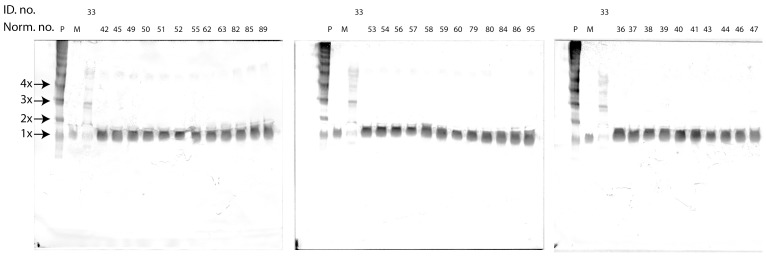
Native PAGE WB analysis of EDTA plasma samples from 34 healthy individuals, and one polymer positive HAE patient (lab. nr. 33: CPDA plasma). “Norm. nr.”. depicts the number ascribed to each healthy individual, “P”, “M” and “ID. no.” depictions as for figure 3.

The polymerization patterns observed in EDTA plasma and CPDA plasma samples were identical (data not shown). No polymer bands were observed when EDTA plasma samples from 34 healthy individuals were analyzed by the native PAGE WB method (Fig. 5). Five repeated freeze/thaw cycles of the EDTA plasma pool had no effect on the native PAGE WB results (data not shown).

### Structural predictions

**Figure 6:**
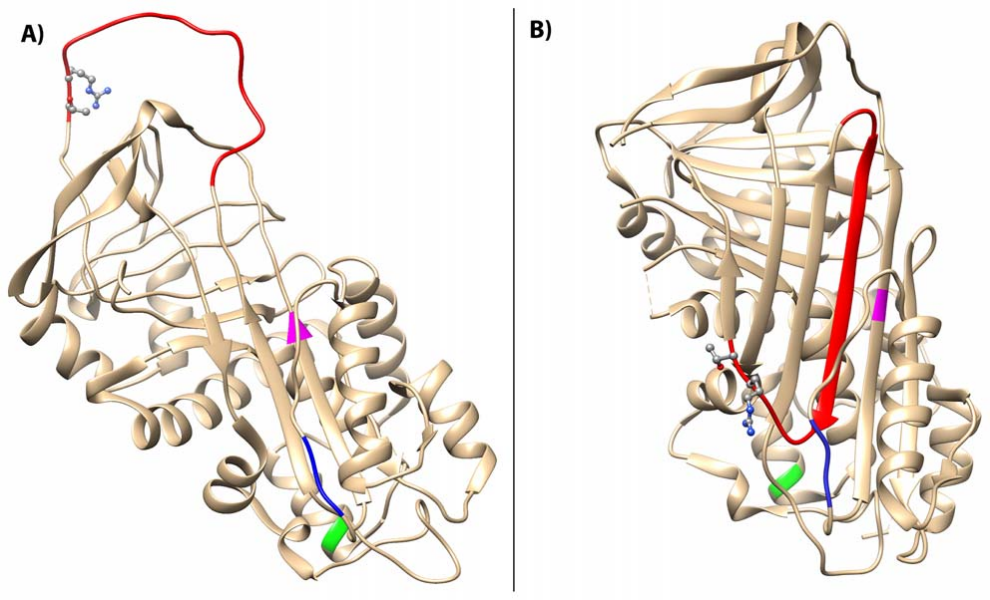
Structural localization of mutations. A) Homology model of the native C1-inh serpin domain (PDB entry 1M6Q). The RCL is depicted in red and the P1–P1′ scissile band (Arg444-Thr445) is shown in ball-and stick representation. Locations of the three mutations are marked as follows Ile271Thr (magenta), Ser258_Pro260del (blue) and Thr167Asn (green). B) Latent C1-inh marked with the three mutation sites giving rise to C1-inh polymerization (PDB entry 20AY).

The locations of the three identified polymerogenic mutations on the C1-inh protein are depicted on two X-ray crystallographic representations of a homology model of native and latent C1-inh form (protein database entry 1M6Q and 2OAY, respectively) (Fig. 6). Mutation number 6 (p.Ile271Thr) is located in strand 3A on beta-sheet A, which is positioned in the proximity to the RCL insertion site. The deletion mutation number 13 (p.Ser258_Pro260del) affects a region related to helix F, which is located just above the site of RCL insertion. Mutation number 21 (p.Thr167Asn) affects helix C located behind the site of RCL insertion.

## Discussion

In the present study we aimed to elucidate whether certain HAE genotypes produced C1-inh polymers identified with a specific monoclonal antibody. All Danish HAE families were tested for a putative polymerized C1-inh phenotype. We demonstrated that C1-inh polymers were present in plasma of six HAE patients in three of 31 HAE families affected by different *SERPING1* mutations.

*In vitro* experiments using recombinant C1-inh strategy have demonstrated that certain C1-inh mutations are prone to polymerization, but these experiments did not demonstrate the presence of polymerized C1-inh in patient plasma [14]. Others have used patient plasma samples subjected to gel filtration or sucrose density gradient centrifugation analysis or C1-inh purified from patient plasma, and the results of these studies advocate for the presence of polymeric C1-inh in patient plasma [8], [12], [13]. However, the presence of C1-inh polymers in untreated patient plasma samples has not previously been demonstrated.

C1-inh polymers were detected in HAE patient plasma samples, with determination of the sizes of the polymers. For these experiments we used a native PAGE WB method, where a high affinity MAb (12-27-15) produced against C1-inh polymers produced using heat denaturation was utilized. Native PAGE separates the various forms of polymerized C1-inh according to their electrophoretic mobility, in contrast to SDS-PAGE, which, as demonstrated in the present study, dissolves polymeric C1-inh into monomers. Therefore, native PAGE is an attractive method to analyze the size distribution of polymerized C1-inh. A limitation to this method is that MAb (12-27-15) was produced against artificially formed C1-inh polymers. This might give rise to false negative results, as in vivo occurring polymers potentially display different epitopes than those observed in C1-inh polymers formed using heat denaturation.

Polymerized C1-inh was prepared by heat denaturation, and was used as marker throughout the experiments. To assure that the molecular weight of the polymer bands observed in native PAGE corresponded to that expected for C1-inh polymers in SDS-PAGE, we conjugated the pC1-inh using the zero-length-linker BS^3^. Using this method, we determined the molecular weight of the BS^3^-conjugated polymers in SDS-PAGE, and subsequently we compared BS^3^-conjugated polymers with pC1-inh in samples on native PAGE. By this approach we confirmed that the molecular weight of pC1-inh, as observed in native PAGE, corresponded to the molecular weight of BS^3^ conjugated polymers (Fig. 1A). Furthermore, we prepared a low molecular weight form of the pC1-inh (LMW pC1-inh), resembling the polymer size observed in patient plasma samples. PC1-inh, LMW pC1-inh and monomeric C1-inh were used as markers in all native PAGE WB, where patient plasma samples were analyzed (Fig. 2). Using this method we detected polymers in patient plasma samples and evaluated the size distribution of the polymeric species.

Patient plasma samples representing each *SERPING1* genotype were analyzed in native PAGE WB. Three genotypes (Mut. no. 6, 13 and 21) gave rise to the presence of C1-inh polymers in the patient plasma samples (Fig. 3). The polymerization pattern of each mutation was unique, but four patients bearing the same mutation (Mut. no. 6) presented with identical polymerization patterns in native PAGE WB (Fig. 4). This observation suggests an association between each *SERPING1* genotype and its corresponding C1-inh protein phenotype in plasma. Polymers were not observed in plasma samples from healthy individuals (Fig. 5).

All patients presenting with C1-inh polymers were previously classified as HAE type I patients in the initial setting. This is an intriguing observation as HAE type I patients per definition only secrete functional non-mutated C1-inh into plasma [22]. It is here worth to note that the classification of HAE patients into type I and II is highly dependent upon the methods used for classification. Usually the C1-inh antigen concentration is measured either by a C1-inh specific enzyme linked immunosorbent assays (ELISA), or by nephelometric analysis. Both methods utilize specific anti-C1-inh antibodies. When C1-inh circulates in its mutated polymeric form, its conformation is dramatically changed, and the anti-C1-inh antibodies used for the classifications do not necessarily recognize the mutated polymerized form. Similar observations were done by Eldering et al. [8], reporting two HAE type I families that also presented with C1-inh polymer as demonstrated by gel filtration.

Mutation number 12 was associated with a low molecular weight C1-inh band (Fig. 3) without a polymerization phenotype. This band probably represents a secreted mutated form of C1-inh, which apparently is not recognized by the anti C1-inh antibodies in the nephelometric methods used for the type I and II classification, as patients carrying this mutation are identified as type I patients [1]. It is important to emphasize that the present native PAGE WB method might suffer from similar problems with regard to recognition of mutated C1-inh. We cannot exclude the possibility that the assay does not recognize all C1-inh polymer forms, as the epitope recognized by our MAb might be masked in specific C1-inh mutations.

The positions in the peptide sequence of the polymerogenic *SERPING1* mutations have been located in the model (Fig. 6). Mutation number 6 (p.Ile271Thr) is located in strand 3A on beta-sheet A, in the region termed the shutter domain. It is well established that mutations affecting the shutter domain of α_1_-antitrypsin lead to polymerization of this serpin [23]. The underlying mechanisms are suggested to involve opening of the beta-sheet A in the mutated serpin, and subsequently the RCL of another α_1_-antitrypsin molecule can insert into this domain with ensuing polymer formation. Mutation number 13 (p.Ser258_Pro260del) results in a deletion in the strand connecting helix F with strand 3 in beta-sheet A. Helix F is positioned in front of the shutter domain, and in this way hinders opening of beta-sheet A, which protects the serpins against polymer formation [24]. We speculate that deletions in the strand connecting helix F and strand 3A of C1-inh result in dislocation of the helix F and opening of beta-sheet A, with subsequent insertion of the RCL from another C1-inh molecule.

Mutation number 21 (p.Thr167Asn) introduces an asparagine residue in helix C of C1-inh, and potentially a new N-glycosylation site in helix C. To our knowledge no mutations affecting helix C in serpins have previously been associated with polymerization. An explanation for this observation is that the mutation may facilitate interactions between helix C and beta-sheet A. Such interactions may possibly result in opening of beta-sheet A and thereby resulting in polymerization.

In this study we only focused on the presence of C1-inh polymers in the plasma of HAE patients, and not on the specific events leading to this observation. Therefore it can only be speculated whether the polymers are assembled in the blood stream, or if they accumulate intracellularly prior to secretion into the blood stream. Additional experiments involving recombinant expression of the polymerogenic mutants are needed to elucidate this question.

The present series of experiments demonstrate that at least six of 75 HAE patients carrying *SERPING1* mutations have C1-inh polymers in plasma. The specific role of C1-inh polymers in the pathophysiology of HAE is still not clear. In addition to the inability of polymers to control target proteases it has been demonstrated that misfolded proteins are potent activators of the kallikrein kinin system [25]. Further experiments are needed to elucidate whether C1-inh polymers present in the plasma from HAE patients, can potentiate formation of bradykinin through activation of the kallikrein kinin system.
